# Alternate day fasting for weight loss in normal weight and overweight subjects: a randomized controlled trial

**DOI:** 10.1186/1475-2891-12-146

**Published:** 2013-11-12

**Authors:** Krista A Varady, Surabhi Bhutani, Monica C Klempel, Cynthia M Kroeger, John F Trepanowski, Jacob M Haus, Kristin K Hoddy, Yolian Calvo

**Affiliations:** 1 Department of Kinesiology and Nutrition, University of Illinois at Chicago, 1919 West Taylor Street, Room 506 F, Chicago, IL 60612, USA

**Keywords:** Alternate day fasting, Calorie restriction, Weight loss, Cholesterol, Blood pressure, Adipokines, Coronary heart disease, Non-obese humans

## Abstract

**Background:**

Alternate day fasting (ADF; ad libitum “feed day”, alternated with 25% energy intake “fast day”), is effective for weight loss and cardio-protection in obese individuals. Whether these effects occur in normal weight and overweight individuals remains unknown. This study examined the effect of ADF on body weight and coronary heart disease risk in non-obese subjects.

**Methods:**

Thirty-two subjects (BMI 20–29.9 kg/m^2^) were randomized to either an ADF group or a control group for 12 weeks.

**Results:**

Body weight decreased (P < 0.001) by 5.2 ± 0.9 kg (6.5 ± 1.0%) in the ADF group, relative to the control group, by week 12. Fat mass was reduced (P < 0.001) by 3.6 ± 0.7 kg, and fat free mass did not change, versus controls. Triacylglycerol concentrations decreased (20 ± 8%, P < 0.05) and LDL particle size increased (4 ± 1 Å, P < 0.01) in the ADF group relative to controls. CRP decreased (13 ± 17%, P < 0.05) in the ADF group relative to controls at week 12. Plasma adiponectin increased (6 ± 10%, P < 0.01) while leptin decreased (40 ± 7%, P < 0.05) in the ADF group versus controls by the end of the study. LDL cholesterol, HDL cholesterol, homocysteine and resistin concentrations remained unchanged after 12 weeks of treatment.

**Conclusion:**

These findings suggest that ADF is effective for weight loss and cardio-protection in normal weight and overweight adults, though further research implementing larger sample sizes is required before solid conclusion can be reached.

## Introduction

Intermittent fasting regimens, particularly alternate day fasting (ADF) protocols, have gained considerable popularity in the past decade. Alternate day fasting involves a “fast day” where individuals consume 25% of energy needs, alternated with a “feed day” where subjects eat ad libitum [1]. Only a handful of studies have been performed to test the effects of ADF on body weight and coronary heart disease (CHD) risk reduction, and almost all of these studies have been undertaken in obese populations (BMI 30–39.9 kg/m^2^) [2-4]. Results from these initial trials indicate that ADF is effective for weight loss (5-6% reductions in body weight) and visceral fat mass loss (5–7 cm reductions in waist circumference) in 8–12 weeks of treatment [2-4]. These reports also suggest that ADF may aid in the retention of lean mass in obese individuals [2-4]. In addition to these favorable body composition changes, improvements in CHD risk have also been noted. For instance, decreases in LDL cholesterol concentrations (20-25%), triacylglycerol concentrations (15-30%), and increases in LDL particle size are often observed with short-term ADF (8–12 weeks) [2-4]. Beneficial changes in blood pressure and adipokine profile (i.e. increases in adiponectin, and decreases in leptin and resistin) have also been reported [2-4]. Taken together, this preliminary work suggests that ADF may be effective for weight loss and CHD risk reduction in *obese* adults.

An important question that remains unresolved is whether the favorable effects of ADF can also be observed in normal weight and overweight populations. Only two human studies [5,6] have tested the effect of ADF on body weight and CHD risk in non-obese subjects. In a study by Heilbronn et al. [5], normal weight men and women (BMI 23 kg/m^2^) participated in an ADF regimen for 3 weeks. Body weight decreased by 2% from baseline, while triacylglycerol concentrations decreased only in men [5]. Contrary to these findings, Halberg et al. [6] demonstrated no change in body weight after 2 weeks of ADF in overweight men (BMI 26 kg/m^2^). While these trials [5,6] lay some groundwork, they are limited by their short durations (2–3 weeks) and their lack of a control group. As such, a longer-term trial (12 weeks) that employs a control group is well warranted.

Accordingly, the present study examined the effect of ADF on body weight, body composition, and CHD risk parameters in both normal weight and overweight adults in a 12-week randomized controlled feeding trial. We hypothesized that ADF would reduce body weight and CHD risk in normal weight and overweight participants, when compared to controls.

### Subjects and methods

#### Subjects

**Figure 1:**
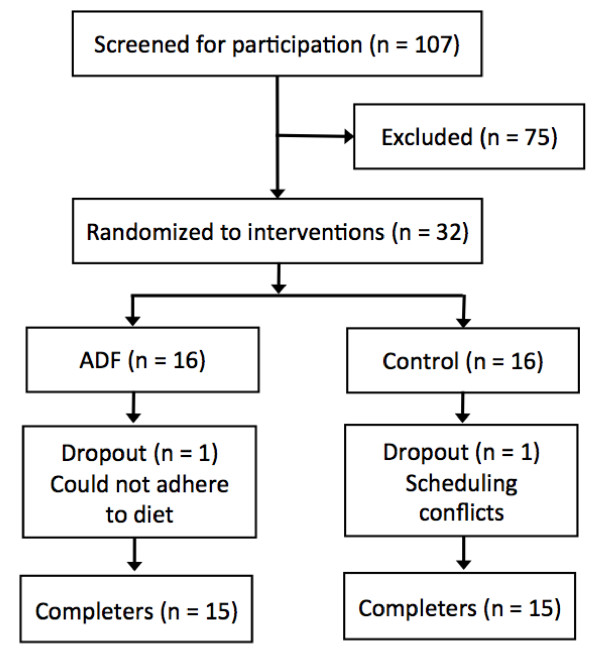
**Study flow chart.** ADF: Alternate day fasting.

Subjects were recruited from the Chicago area by means of advertisements placed around the University of Illinois, Chicago campus. A total of 107 individuals expressed interest in the study, but only 32 were recruited to participate after screening via a preliminary questionnaire and BMI assessment (Figure 1). Inclusion criteria were as follows: BMI between 20 and 29.9 kg/m^2^; age between 35 and 65 years; pre-menopausal or post-menopausal (absence of menses for more than 2 years); lightly active (< 3 h/week of light intensity exercise at 2.5 to 4.0 metabolic equivalents (METs) for 3 months prior to the study); weight stable for 3 months prior to the beginning of the study (< 4 kg weight loss or weight gain); non-diabetic; no history of cardiovascular disease; non-smoker; and not taking weight loss, lipid- or glucose-lowering medications. The experimental protocol was approved by the University of Illinois, Chicago, Office for the Protection of Research Subjects, and all research participants gave their written informed consent to participate in the trial. The research protocol was in compliance with the Helsinki Declaration.

### Study design

#### Experimental design

A 12-week, randomized, controlled, parallel-arm feeding trial was implemented as a means of testing the study objectives. Subjects were randomized by KAV by way of a stratified random sample. Subjects were first divided into strata based on sex (M/F), age (35–50 y/51-65 y), and BMI (20–24.9 kg/m^2^/ 25–29.9 kg/m^2^), and then subjects from each stratum were randomized 1:1 into either the ADF or control group (Figure 1).

#### Diet protocol

During the dietary intervention period, ADF subjects consumed 25% of their baseline energy needs on the fast day (24 h), and then ate ad libitum on each alternating feed day (24 h). Energy needs for each subject were determined by the Mifflin equation [7]. The feed and fast days began at midnight each day, and all fast day meals were consumed between 12.00 pm and 2.00 pm to ensure that each subject was undergoing the same duration of fasting. ADF subjects were provided with meals on each fast day (ranging from 400–600 kcal), and ate ad libitum at home on the feed day. All ADF fast day meals were prepared in the metabolic kitchen of the Human Nutrition Research Center (HNRU) at the University of Illinois, Chicago. Fast day meals were provided as a 3-day rotating menu, and were formulated based on the American Heart Association (AHA) guidelines (30% kcal from fat, 15% kcal from protein, 55% kcal from carbohydrate) [8]. All meals were consumed outside of the research center. ADF subjects were permitted to consume energy-free beverages, tea, coffee, and sugar-free gum, and were encouraged to drink plenty of water. Control subjects were permitted to eat ad libitum every day, and were not provided with meals from the research center.

#### Blood collection protocol

Twelve-hour fasting blood samples were collected between 6.00 am and 9.00 am at baseline (week 1) and post-treatment (week 12). Participants were instructed to avoid exercise, alcohol, and coffee for 24 h before each visit. Blood was centrifuged for 15 min at 520 × g and 4°C to separate plasma from RBCs, and was stored at -80°C until analysed.

### Analyses

#### Energy intake on feed and fast days

During the 12-week diet intervention, subjects in the ADF group were instructed to eat only the foods provided on each fast day. To assess energy intake on the fast days, ADF subjects were asked to report any *extra* food items consumed (i.e. those not provided) using an “Extra food log”. Additionally, subjects were instructed to return any leftover food items to the HNRU for weighing. To assess energy intake on the feed days, ADF and control subjects were asked to complete a 3-day food record on 2 feed days during the week, and on 1 feed day during the weekend, at week 1 and 12. At baseline, the Research Dietician provided 15 min of instruction to all participants on how to complete the food records. These instructions included verbal information and detailed reference guides on how to estimate portion sizes and record food items in sufficient detail to obtain an accurate estimate of dietary intake. All dietary information from the food logs/records was entered into the food analysis program, Nutritionist Pro (version 5, Axxya Systems, Stafford, TX) to assess energy intake.

#### Hunger, satisfaction, and fullness

A validated visual analog scale (VAS) was used to measure hunger, fullness, and satisfaction with the ADF diet [9]. The scale was completed on 3 fast days (before bedtime) at week 1 and 12. In brief, the VAS consisted of 100-mm lines, and subjects were asked to make a vertical mark across the line corresponding to their feelings from 0 (not at all) to 100 (extremely) for hunger, satisfaction, or fullness. Quantification was performed by measuring the distance from the left end of the line to the vertical mark.

#### Weight loss and body composition

Body weight was assessed to the nearest 0.25 kg at the beginning of every week without shoes and in light clothing using a balance beam scale at the HNRU (HealthOMeter, Sunbeam Products, Boca Raton, FL). BMI was assessed as kg/m^2^. Body composition (fat mass and fat free mass) was measured using dual x-ray absorptiometry (DXA) (Hologic QDR 4500 W, Hologic Inc., Waltham, MA).

#### Lipid coronary heart disease risk factors

Plasma total cholesterol, HDL-cholesterol, and triacylglycerol concentrations were measured in duplicate using enzymatic kits (Biovision Inc., Moutainview, CA) at week 1 and 12. The concentration of LDL-cholesterol was calculated using the Friedewald, Levy and Fredrickson equation. LDL particle size was measured by linear polyacrylamide gel electrophoresis (Quantimetrix Lipoprint System, Redondo Beach, CA, USA) at week 1 and 12 [10,11]. High-resolution 3% polyacrylamide gel tubes were used for electrophoresis. Briefly, 25 μL of sample was mixed with 200 μL of liquid loading gel containing Sudan black, and added to the gel tubes. After photopolymerization at room temperature for 30 min, samples were electrophoresed for 1 h (3 mA/gel tube). Lipoware computer software (Quantimetrix, Redondo Beach, CA, USA) was then used to divide LDL into small (<255 Å), medium (255–260 Å), and large (>260 Å) particles, and to assess mean LDL particle size [10]. The intra-assay coefficients of variation (CV) for total cholesterol, HDL cholesterol, triacylglycerol, and LDL particle size were 3.6%, 4.8%, 2.5%, and 4.1%, respectively.

#### Non-lipid coronary heart disease risk factors

All measurements were taken at week 1 and 12. Blood pressure was measured in triplicate with the subject in a seated position after a 10-min rest. C-reactive protein (CRP) was measured in duplicate using Immulite 1000 High Sensitivity CRP kits (Diagnostic Products Corporation, Los Angeles, CA). Plasma homocysteine measurements were carried out in duplicate using HPLC with fluorometric detection. Adiponectin, leptin and resistin were measure by ELISA (R&D Systems, Minneapolis, MN). The intra-assay coefficients of variation (CV) for CRP, homocysteine, adiponectin, leptin, and resistin were 5.0%, 4.3%, 3.3%, 3.0%, and 4.7%, respectively.

### Statistics

Results are presented as means ± standard error of the mean (SEM). Tests for normality were included in the model. No variables were found to be not normal. Differences between groups at baseline were tested by independent samples *t*-test. Within-group changes from week 1 to 12 were tested by a paired *t*-test. Between-group differences were tested by an independent samples *t*-test. Sample size was calculated assuming a 10% change in LDL-cholesterol concentrations in the ADF group, with a power of 80% and an alpha risk of 5%. P-values of < 0.05 were considered significant. Data were analyzed by using SPSS software (version 21.0 for Mac OS X; SPSS Inc., Chicago, IL).

## Results

### Subject baseline characteristics and dropouts

Thirty-two subjects commenced the study, with 30 completing the entire 12-week trial (Figure 1). After loss due to dropouts, the remaining subjects in each intervention group were as follows: ADF (n = 15) and control (n = 15). Baseline characteristics of the subjects who completed the trial are presented in Table 1. There were no significant differences at the beginning of the study between groups for age, sex, ethnicity, body weight, body composition, height or BMI.

**Table 1:** Subject characteristics at baseline

	**ADF-ALL**	**Control**	**P-value**^**1**^
n	15	15	
Age (y)	47 ± 3	48 ± 2	0.18
Sex (M/F)	5 / 10	3 / 12	0.44
Ethnicity (n)			
African American	5	8	0.30
Caucasian	8	6	
Hispanic	2	1	
Other	0	0	
Body weight (kg)	77 ± 3	77 ± 3	0.79
Fat mass (kg)	26 ± 2	27 ± 1	0.13
Fat free mass (kg)	51 ± 3	50 ± 3	0.78
Height (cm)	171 ± 3	170 ± 2	0.71
BMI (kg/m^2^)	26 ± 1	26 ± 1	0.75

Values reported as mean ± SEM. *ADF*: Alternate day fasting, *BMI*: Body mass index, *F*: Female, *M*: Male.

^1^P-value between groups at baseline: Independent samples *t*-test.

### Energy intake, hunger, satisfaction and fullness

Energy intake, hunger, satisfaction, and fullness are reported in Table 2. At baseline, there were no differences between the ADF and control groups for feed day energy intake. From week 1 to 12 of the study, energy intake remained constant on both feed and fast days in the ADF group. Adherence to the fast day protocol was high in the ADF group (98 ± 5%). Hunger levels were moderate as baseline, and did not change by week 12 in either group. Satisfaction and fullness increased (P < 0.01) from baseline to post-treatment in the ADF group, with no change in the control group.

**Table 2:** Energy intake, hunger, satisfaction and fullness during the 12-week study

	**Intervention**	**Week 1**^**1**^	**Week 12**	**P-value**^**2**^	**P-value**^**3**^	**Change**^**4**^	**P-value**^**5**^
Feed day energy intake (kcal/d)	ADF	1874 ± 136	1856 ± 229	0.93	0.92	-18 ± 186	0.50
	Control	1873 ± 243	1790 ± 286	0.71		-82 ± 75	
Fast day energy intake (kcal/d)	ADF	482 ± 19	489 ± 20	0.74		7 ± 5	
Hunger (mm)	ADF	5 ± 1	4 ± 1	0.44	0.56	-1 ± 1	0.38
	Control	5 ± 1	5 ± 1	0.46		0 ± 1	
Satisfaction (mm)	ADF	4 ± 1	7 ± 1	<0.01	0.81	3 ± 1	0.22
	Control	6 ± 1	7 ± 1	0.34		1 ± 1	
Fullness (mm)	ADF	2 ± 1	4 ± 1	<0.01	0.78	2 ± 1	0.01
	Control	6 ± 1	6 ± 1	0.57		0 ± 1	

Values reported as mean ± SEM. *ADF*: Alternate day fasting.

^1^Baseline values were not significantly different between intervention groups for any parameter: Independent samples *t*-test.

^2^P-value between week 1 and week 12: Paired *t*-test.

^3^P-value between groups at week 12: Independent samples *t*-test.

^4^Absolute change between week 1 and week 12 values.

^5^P-value between groups for absolute change: Independent samples *t*-test.

### Weight loss and body composition

**Figure 2:**
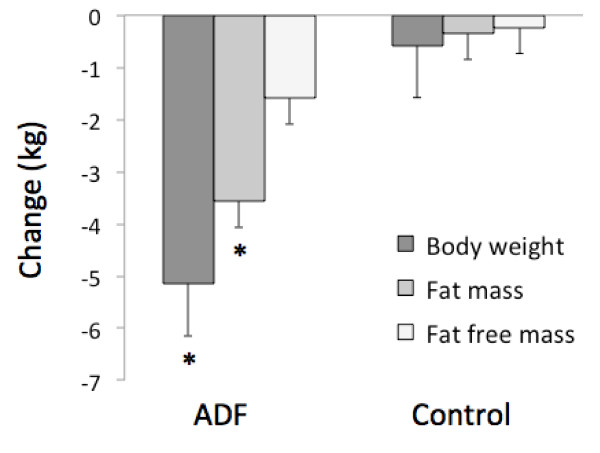
**Body weight and body composition changes at week 12.** Values reported as mean ± SEM. ADF: Alternate day fasting. *Body weight and fat mass significantly different (P < 0.001) from the control group at week 12 (Independent samples *t*-test). No difference between groups for fat free mass at week 12 (Independent samples *t*-test).

Changes in body weight and body composition are displayed in Figure 2. Body weight decreased (P < 0.001) by 5.2 ± 0.9 kg (6.5 ± 1.0%) in the ADF group, relative to the control group at week 12. Fat mass was reduced (P < 0.001) by 3.6 ± 0.7 kg, and fat free mass did not change, versus controls.

### Lipid coronary heart disease risk factors

Changes in plasma lipids and LDL particle size are reported in Table 3. Total cholesterol concentrations decreased (P < 0.01) in the ADF group when post-treatment values were compared to baseline. However, changes in total cholesterol levels were not significantly different from controls at week 12. LDL cholesterol concentrations were reduced (P = 0.01) within the ADF group, but no significant between-group differences were noted. HDL cholesterol concentrations remained unchanged throughout the trial. Triacylglycerol concentrations decreased (P = 0.01) in the ADF group relative to controls at week 12. Non-HDL cholesterol levels were reduced (P < 0.01) within the ADF group, but no significant between-group differences were observed. LDL particle size increased (P < 0.01) in the ADF group relative to controls by the end of the study.

**Table 3:** Lipid coronary heart disease risk factor changes during the 12-week study

	**Intervention**	**Week 1**^**1**^	**Week 12**	**P-value**^**2**^	**P-value**^**3**^	**Change**^**4**^	**P-value**^**5**^
Total cholesterol (mg/dl)	ADF	201 ± 9	175 ± 12	<0.01	0.12	-26 ± 6	0.50
	Control	211 ± 11	202 ± 9	0.54		-9 ± 5	
LDL cholesterol (mg/dl)	ADF	118 ± 9	99 ± 9	0.01	0.82	-18 ± 6	0.29
	Control	128 ± 10	119 ± 6	0.08		-9 ± 4	
HDL cholesterol (mg/dl)	ADF	56 ± 3	54 ± 4	0.49	0.77	-2 ± 3	0.51
	Control	57 ± 2	58 ± 4	0.83		1 ± 2	
Triacylglycols (mg/dl)	ADF	109 ± 13	87 ± 9	0.06	0.01	-22 ± 11	0.22
	Control	108 ± 18	118 ± 19	0.34		10 ± 7	
Non-HDL cholesterol (mg/dl)	ADF	149 ± 11	124 ± 12	<0.01	0.54	-25 ± 5	0.96
	Control	153 ± 12	144 ± 10	0.79		-9 ± 5	
LDL particle size (Å)	ADF	254 ± 1	258 ± 2	<0.01	<0.01	4 ± 1	0.13
	Control	252 ± 2	250 ± 3	0.16		-2 ± 1	

Values reported as mean ± SEM. ADF: Alternate day fasting.

^1^Baseline values were not significantly different between intervention groups for any parameter: Independent samples *t*-test.

^2^P-value between week 1 and week 12: Paired *t*-test.

^3^P-value between groups at week 12: Independent samples *t*-test.

^4^Absolute change between week 1 and week 12 values.

^5^P-value between groups for absolute change: Independent samples *t*-test.

### Non-lipid coronary heart disease risk factors

Changes in blood pressure, homocysteine, CRP, and adipokines are shown in Table 4. Systolic and diastolic blood pressure decreased (P < 0.05) within the ADF group, but no significant between-group differences were noted. CRP decreased (P = 0.01) in the ADF group relative to controls at week 12. Plasma adiponectin increased (P < 0.01) while leptin decreased (P = 0.03) in the ADF group versus controls by the end of the study. Plasma homocysteine and resistin concentrations remained unchanged after 12 weeks of treatment.

**Table 4:** Non-lipid coronary heart disease risk factor changes during the 12-week study

	**Intervention**	**Week 1**^**1**^	**Week 12**	**P-value**^**2**^	**P-value**^**3**^	**Change**^**4**^	**P-value**^**5**^
Systolic BP (mm Hg)	ADF	124 ± 4	117 ± 4	0.02	0.85	-7 ± 2	0.51
	Control	119 ± 3	120 ± 4	0.67		1 ± 3	
Diastolic BP (mm Hg)	ADF	78 ± 3	72 ± 2	0.03	0.05	-6 ± 2	0.17
	Control	82 ± 4	84 ± 5	0.28		2 ± 6	
Homocysteine (umol/dl)	ADF	9 ± 1	9 ± 1	0.37	0.50	0 ± 1	0.21
	Control	9 ± 1	9 ± 1	0.86		0 ± 1	
C-reactive protein (mg/L)	ADF	2 ± 1	1 ± 1	0.29	0.01	-1 ± 1	0.01
	Control	1 ± 1	1 ± 1	0.78		0 ± 1	
Adiponectin (ng/ml)	ADF	10728 ± 1251	11401 ± 1197	0.58	0.15	672 ± 1191	<0.01
	Control	11350 ± 1369	10509 ± 1316	0.26		-842 ± 623	
Leptin (ng/ml)	ADF	25 ± 4	15 ± 3	0.04	0.55	-10 ± 3	0.03
	Control	22 ± 7	18 ± 6	0.07		-4 ± 3	
Resistin (ng/ml)	ADF	18 ± 3	15 ± 4	0.15	0.57	-3 ± 3	0.20
	Control	23 ± 4	21 ± 4	0.26		-2 ± 2	

Values reported as mean ± SEM. *ADF*: Alternate day fasting.

^1^Baseline values were not significantly different between intervention groups for any parameter: Independent samples *t*-test.

^2^P-value between week 1 and week 12: Paired *t*-test.

^3^P-value between groups at week 12: Independent samples *t*-test.

^4^Absolute change between week 1 and week 12 values.

^5^P-value between groups for absolute change: Independent samples *t*-test.

## Discussion

This study shows, for the first time, that ADF is an effective strategy for moderate weight loss (6%) in normal weight and overweight subjects. This diet strategy may also have cardio-protective effects in non-obese subjects, by way of lowering triacylglycerols, CRP and leptin, while increasing LDL particle size and adiponectin concentrations.

The primary goal of this study was to determine if non-obese individuals could benefit from ADF in terms of weight loss. Previous ADF studies implementing non-obese subjects report inconsistent findings [5,6]. While one study demonstrated decreases in body weight of 2% from baseline after 3 weeks of ADF [5], another study showed no effect after 2 weeks of diet [6]. The limited amount of weight loss reported previously is undoubtedly a factor of the short trial durations implemented [5,6]. Thus, we wanted to determine if the degree of weight loss could be amplified if the trial duration was extended to 12 weeks. We show here that normal weight and overweight subjects can indeed benefit from ADF, as body weight was reduced by 6% (5 kg) by the end of the trial. This degree of weight loss in non-obese participants is similar to what has been reported for obese individuals undergoing ADF [2-4]. For instance, Bhutani et al. [4] demonstrated 5% (5 kg) weight loss after 12 weeks of ADF in obese men and women. In line with these findings, Klempel et al. [3] and Varady et al. [2] report 5-6% (5–6 kg) weight loss after 8 weeks of treatment in obese subjects. Thus, ADF may produce a mean rate of weight loss of approximately 0.5 kg/week, independent of the starting weight or BMI class of the subject. Fat free mass was also retained after 12 weeks of ADF in non-obese individuals. This finding is similar to what has been reported in previous short-term studies of ADF [2-4]. As such, the beneficial preservation of fat free mass observed in obese individuals [2-4] may be replicated in non-obese subjects participating in ADF protocols.

Our findings also indicate that normal weight and overweight subjects have no problem adhering to the fast day protocol for 12 weeks. Dietary adherence was very high at baseline (98%), and did not wane over the course of the study. It should be noted, however, that one normal weight subject dropped out of the trial due to an inability to adhere to the diet. Notwithstanding, our dropout rate was still less than 10%, which is similar to the dropout rate of studies performed in obese individuals [2-4]. Complementary to previous reports [12,13], there was very little or no hyperphagic response on the feed day in response to the lack of food on the fast day. This lack of hyperphagia allowed for overall energy restriction to remain high throughout the study, and undoubtedly contributed to the sizeable degree of weight loss observed here. As for eating behaviors, perceived hunger was moderate at baseline and did not change by week 12. This is contrary to findings in obese participants, which consistently show declines in hunger after 8–12 weeks of ADF [11,12]. Dietary satisfaction and feelings of fullness, on the other hand, increased from baseline to post-treatment. These increases in satisfaction and fullness have also been noted in obese subjects [11,12], and may play a role in long-term adherence to the diet.

The cardio-protective effects of ADF were also examined. Reductions in triacylglycerol concentrations (20%) were noted after 12 weeks of ADF. LDL particle size also increased post-treatment (4 Å from baseline). These changes in lipid risk factors are in line with what has been reported for obese ADF subjects [14,15]. In two recent ADF studies, triacylglycerols decreased by 15% and LDL particle size increased by 2–3 Å after 8 weeks of treatment in obese men and women [14,15]. Thus, ADF may improve plasma lipids to the same extent in non-obese subjects as it does in obese subjects. Additional vascular benefits, including decreases in circulating leptin and CRP concentrations, in conjunction with increases in adiponectin, were also noted in non-obese subjects undergoing ADF. As for HDL cholesterol, homocysteine, and resistin concentrations, no effect was observed. This lack of effect is not surprising as these CHD risk parameters are generally only improved with >10% weight loss [16-18].

It will be of interest in future studies to determine how alterations in macronutrient intake on the fast day may affect weight loss and cardiovascular outcomes. For instance, it has been well established that Mediterranean [19] and certain low-carbohydrate diets [20] help to maintain a healthy body weight and reduce CHD risk. Whether further reductions in body weight and CHD risk would occur if ADF were combined with Mediterranean or low-carbohydrate diets, undoubtedly warrants investigation.

A couple of adverse events were reported during the study. Two subjects experienced mild headaches during week 1 of the trial, which may or may not be related to dietary treatment. One other subject reported constipation during week 1 and 2 of the trial. The subject was advised to consume more fruits and vegetables on feed days, and the constipation subsided by week 3 of the dietary intervention period.

This study has several limitations. First and foremost, it must be acknowledged that this pilot study was originally designed to compare the effects of ADF in normal weight versus overweight individuals on body weight and CHD risk. Due to a low recruitment rate, we were only able to recruit n = 8 subjects into the normal weight group and n = 8 subjects into the overweight group. In view of this, we decided to combine the normal weight and overweight groups into one group to increase sample size. This post hoc change should be taken into consideration when interpreting the findings of this paper. Secondly, physical activity was not assessed throughout the trial, thus the degree of weight loss associated with increased energy expenditure from exercise is not known. Thirdly, the sample size of each group was small (n = 15). Thus, this study may not be adequately powered to detect changes in certain CHD risk parameters (e.g. resistin). Fourthly, this study employed food records to assess dietary intake/adherence. It is well known that overweight subject underreport food intake by ~30% [21,22]. Thus, our findings for the hyperphagic response on the feed day may be inaccurate.

In summary, these preliminary findings suggest that ADF is a viable weight loss strategy for normal weight and overweight individuals wishing to lose a moderate amount of weight (5–6 kg) within a relatively short period of time (12 weeks). This diet may also help lower CHD risk in non-obese individuals, though further investigation is warranted to confirm these effects. It should also be noted that the purpose of this paper is to report pilot feasibility findings. It is our hope that this preliminary data will be utilized to design larger-scale longer-term trials with similar objectives, in normal weight and overweight participants undergoing ADF.

## Competing interest

The authors have no conflicts of interest to report.

## Authors’ contributions

KAV designed the experiment, analyzed the data, and wrote the manuscript. SB, MCK, CMK, and JFT assisted with the conduction of the clinical trial and performed the laboratory analyses. JMH assisted with the data analyses and the preparation of the manuscript. KKH and YC assisted with the laboratory analyses. All authors read and approved the final manuscript.
